# The structure of a prokaryotic viral envelope protein expands the landscape of membrane fusion proteins

**DOI:** 10.1038/s41467-019-08728-7

**Published:** 2019-02-19

**Authors:** Kamel El Omari, Sai Li, Abhay Kotecha, Thomas S. Walter, Eduardo A. Bignon, Karl Harlos, Pentti Somerharju, Felix De Haas, Daniel K. Clare, Mika Molin, Felipe Hurtado, Mengqiu Li, Jonathan M. Grimes, Dennis H. Bamford, Nicole D. Tischler, Juha T. Huiskonen, David I. Stuart, Elina Roine

**Affiliations:** 1 Division of Structural Biology, Wellcome Centre for Human Genetics, University of Oxford, Oxford, OX3 7BN UK; 2 Diamond Light Source Limited, Harwell Science and Innovation Campus, Didcot, OX11 0DE UK; 3 Fundación Ciencia & Vida, Laboratorio de Virología Molecular, Avenida Zañartu 1482, 7780272 Ñuñoa, Santiago Chile; 4 Department of Biochemistry and Developmental Biology, University of Helsinki, Haartmaninkatu 8, 00014 Helsinki, Finland; 5 Thermo Fisher Scientific, Achtseweg Noorg 5, 5651 GG Eindhoven, The Netherlands; 6 Helsinki Institute of Life Science HiLIFE, Institute of Biotechnology, University of Helsinki, Viikinkaari 5, 00014 Helsinki, Finland; 7 Molecular and Integrative Biosciences Research Program, University of Helsinki, Viikinkaari 1, 00014 Helsinki, Finland; 8 Helsinki Institute of Life Science HiLIFE and Molecular and Integrative Biosciences Research Program, University of Helsinki, Viikinkaari 1, 00014 Helsinki, Finland; 9 Present Address: School of Life Sciences, Tsinghua University, 100084 Beijing, China

## Abstract

Lipid membrane fusion is an essential function in many biological processes. Detailed mechanisms of membrane fusion and the protein structures involved have been mainly studied in eukaryotic systems, whereas very little is known about membrane fusion in prokaryotes. Haloarchaeal pleomorphic viruses (HRPVs) have a membrane envelope decorated with spikes that are presumed to be responsible for host attachment and membrane fusion. Here we determine atomic structures of the ectodomains of the 57-kDa spike protein VP5 from two related HRPVs revealing a previously unreported V-shaped fold. By Volta phase plate cryo-electron tomography we show that VP5 is monomeric on the viral surface, and we establish the orientation of the molecules with respect to the viral membrane. We also show that the viral membrane fuses with the host cytoplasmic membrane in a process mediated by VP5. This sheds light on protein structures involved in prokaryotic membrane fusion.

## Abstract (web summary)

Lipid membrane fusion is an essential function in many biological processes but little is known about membrane fusion in prokaryotes. The authors here study how haloarchaeal pleomorphic viruses (HRPVs) infect archaeal hosts. The structure-function analysis of the spike proteins shed light on prokaryotic membrane fusion.

## Introduction

Lipid membrane fusion is usually driven by membrane-associated proteins that confer specificity and overcome the energy barriers related to the fusion process^1^. There is a wealth of information about the mechanisms and the protein structures involved in eukaryotic membrane fusion. In particular, extensive studies of enveloped viruses infecting eukaryotes have led to their assignment into three structural classes I–III. In contrast, very few examples of enveloped viruses that infect prokaryotes have been characterized. A notable example is the enveloped bacteriophage φ6, which fuses with the outer membrane of its Gram-negative host by a structurally unknown mechanism^2^.

Haloarchaeal pleomorphic viruses (HRPVs) infect archaeal hosts living in hypersaline environments. The newly established family *Pleolipoviridae* includes several HRPVs with Halorubrum pleomorphic virus 1 (HRPV-1) as the typical member^3^. The virions contain two major protein species, a spike protein (VP4 and VP4-like proteins) protruding from the membrane surface and a smaller membrane associated protein (VP3 and VP3-like proteins) residing mostly inside the virion^4,5^. The genomes of pleolipoviruses consist of either single- or double-stranded DNA molecules which are not associated with any protein^5,6^. The VP4 spike protein of HRPV-1 is a type I transmembrane protein (with an N-terminal signal peptide sequence of ~33 amino acids)^4^, N-glycosylated with a pentasaccharide containing legionaminic acid as the terminal residue and suggested to be involved in the host recognition^7^ whereas for other pleolipovirus spike proteins, glycan modifications have not been detected.

In the absence of atomic structures for the spike proteins from enveloped viruses infecting prokaryotes, it is unknown whether they contain fusion proteins that are similar to those characterized in eukaryotic viruses. Pleolipovirus VP4-like surface spike proteins are good candidates to be involved in membrane fusion^5^, however their structure and function remains to be established. The structure on the native virion and the distribution of the VP4-like proteins on the virion envelope also remain unknown.

Here we describe crystallographic structures of VP4-like spike proteins from two pleolipoviruses, HRPV-2 and HRPV-6, showing that these proteins have a unique fold, different from class I–III fusion proteins. By cryo-electron tomography utilizing Volta phase plates we show that the spike protein of HRPV-6 is monomeric on the surface of the virion. Fluorescence-based assays demonstrate that the spikes induce fusion of the viral membrane with the host cell membrane. In addition, we provide preliminary evidence that the membrane fusion may be preceded by a conformational change in the spikes. Together, our structural and functional analysis of the VP4-like spike proteins and virions allows us to suggest a model of prokaryotic virus-cell membrane fusion.

## Results

### Pleolipovirus spike structures reveal a V-shaped fold

**Fig. 1:**
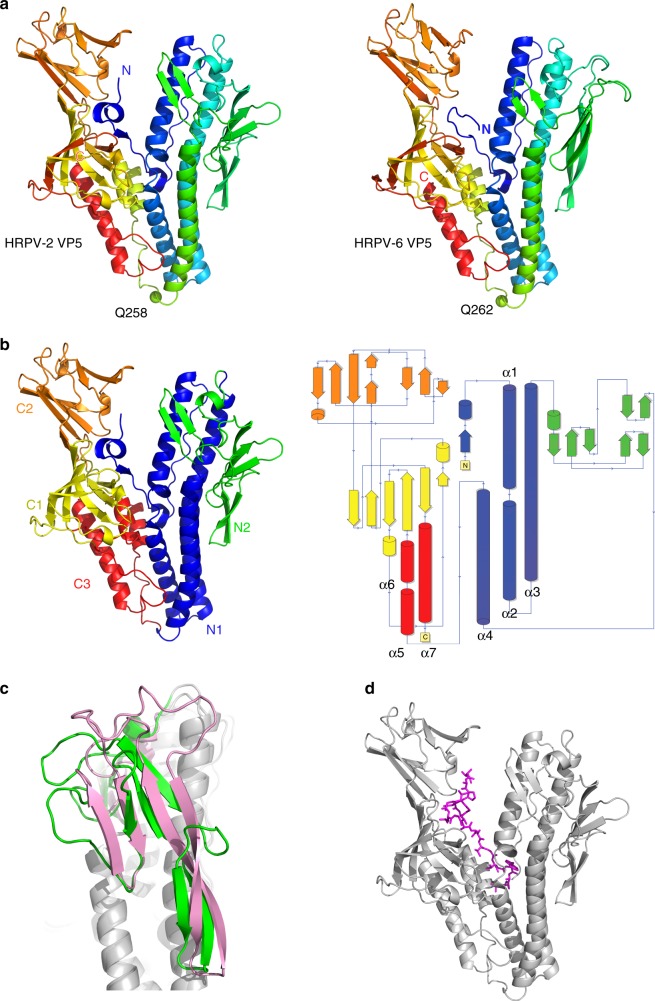
The overall fold of VP5. **a** Cartoon representation of HRPV-2 VP5 (left) and HRPV-6 VP5 (right) coloured from the N-terminal (blue) to the C-terminal (red). Residues Q258 and Q262 connecting the N- and C-terminal domains are shown as green spheres. **b** Cartoon and topology representation of HRPV-2 VP5 coloured by domains. **c** Structural differences between the N2 domains of superposed HRPV-2 VP5 (green) and HRPV-6 VP5 (pink). **d** Localization of the potential fusion peptide depicted as magenta sticks within HRPV-2 VP5 shown as grey cartoon

In order to characterize the VP4-like fusion proteins (termed VP5 in these viruses) from HRPV-2 and HRPV-6, we purified them from infection-competent virions using either detergent (HRPV-2) or protease digestion (HRPV-6). After protein purification, we determined the structures of the HRPV-2 and HRPV-6 VP5 ectodomains (residues 1-498 out of 533 residues and 9-498 out of 537 residues, Supplementary Figure 1) at 2.5 and 2.7 Å resolution, respectively, by X-ray crystallography (Table 1). The two proteins, which are 67% identical in amino acid sequence (Supplementary Figure 1), share a highly conserved V-shaped fold (r.m.s.d. for 453 corresponding Cα atoms: 1.6 Å) (Fig. 1a). The structure is composed of two elongated domains roughly equal in size and linked by a single residue (Q258 in HRPV-2 and Q262 in HRPV-6) (Fig. 1a). The N-terminal domain comprises two subdomains (N1 and N2) while the C-terminal domain includes three subdomains (C1–C3) (Fig. 1b).

**Table 1:** X-ray data collection and refinement details

	HRPV-6 VP5	HRPV-2 VP5
Data collection		
Space group	*P*6_5_22	*P*2_1_2_1_2_1_
Cell dimensions		
* a*, *b*, *c* (Å)	114.3, 114.3, 445.2	48.1, 93.2, 121.8
*α*, *β*, *γ* (°)	90, 90, 120	90, 90, 90
Resolution (Å)	90.45–2.69 (2.76–2.69)^a^	46.61–2.46 (2.52–2.46)^a^
* R*_merge_	16.5 (198.3)	12.6 (40.0)
* I* / σ*I*	17.4 (1.8)	10.2 (2.2)
Completeness (%)	99.9 (99.8)	81.4 (36.3)
Redundancy	25.8 (22.5)	4.6 (2.9)
CC1/2	0.99 (0.75)	0.98 (0.72)
Refinement		
Resolution (Å)	82.35–2.69	81.25–2.46
No. of reflections		
* R*_work_ / *R*_free_	0.218/0.233	0.224/0.241
No. of atoms		
Protein	5493	3740
Ligand/ion	25	18
Water	196	75
* B*-factors (Å^2^)	76.2	39.4
R.m.s. deviations		
Bond lengths (Å)	0.009	0.008
Bond angles (°)	1.1	1.0

^a^Values in parentheses are for highest-resolution shell. Number of crystals: 1

The N-terminal subdomain, N1, contains a bundle of four α-helices, one of which, α3, spans more than 40 residues (Fig. 1b). Interacting with helix α3 of the helix bundle, subdomain N2 is composed of three small β-sheets of two or three strands each. Subdomain N2 is the least conserved between HRPV-2 and HRPV-6 and amongst other pleolipoviruses (Fig. 1c; Supplementary Figure 1).

Previously we have determined the N-terminal sequences of the ectodomains of both HRPV-2 and HRPV-6 VP5 proteins^6^. The first twenty N-terminal residues of both VP5s are hydrophobic and predicted, by several programs for analysing hydrophobic regions of proteins (see Methods), to form a transmembrane helix, indicating the preference of this region to partition into membranes. Interestingly, this hydrophobic segment is shielded from the solvent in both crystal structures by the C-terminal domain. Such protection is typical for fusion peptides or fusion loops in the pre-fusion forms of class I and II fusion proteins^8^, where the shielding is conferred by another chain, either of the same or different protein species.

One of the C-terminal subdomains is α-helical and the other two are formed of β-strands (Fig. 1b). Surprisingly, the entire C-terminal domain is disordered in one of the two molecules of the asymmetric unit of HRPV-6 VP5. Although there is space in the crystal lattice to accommodate this disordered domain, the bulk of adjacent molecules prevents it from adopting the same orientation with respect to the N-terminal domain seen in the other copies of VP5. It appears that the N- and C-terminal domains can flex with respect to each other in the face of crystal packing forces.

The last 20 residues of both HRPV-2 and HRPV-6 VP5s are predicted to form a transmembrane helix^9^. Analysis of proteinase K treated HRPV-6 particles by LC-MS/MS resulted in the identification of several peptides with core sequence FGVPGEVAV representing part of the predicted C-terminal transmembrane domain (Supplementary Figure 2). In contrast, HRPV-2 VP5 was released intact from the virion by detergent treatment. However, the last 35 residues are not visible in the electron density.

### VP5 is monomeric on the virion surface

**Fig. 2:**
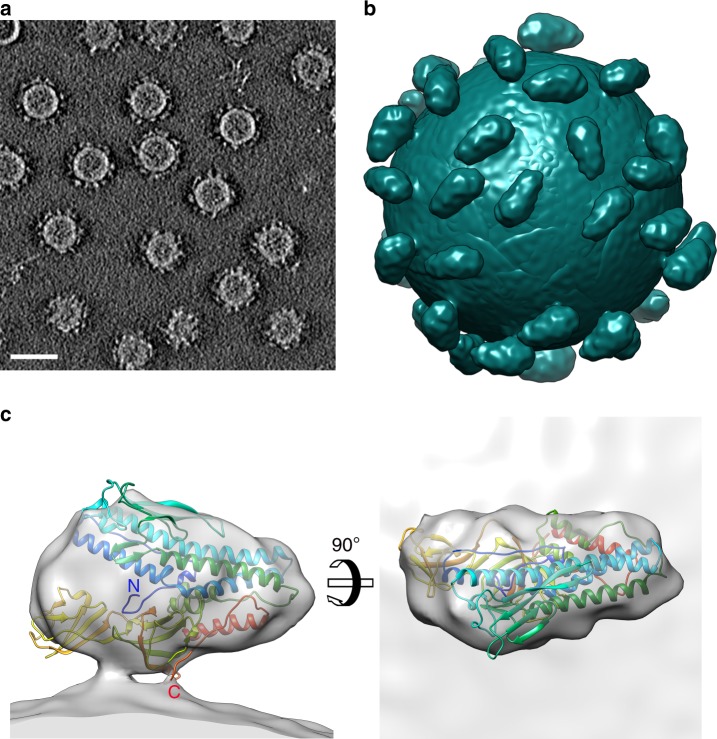
Cryo-electron tomography studies of HRPV-6. **a** A slice through a 3D tomogram of HRPV‐6 reconstructed from tilt series data collected using a Volta phase plate. The scale bar corresponds to 40 nm. **b** Reconstruction of an HRPV-6 virion. 41 VP5 spikes were found from a representative HRPV-6 virion by manual picking. The EM density was projected onto the aligned positions and orientations of these spikes. **c** VP5 crystallographic structure fitted into its density solved by cryo-electron tomography and subtomogram averaging

**Fig. 3:**
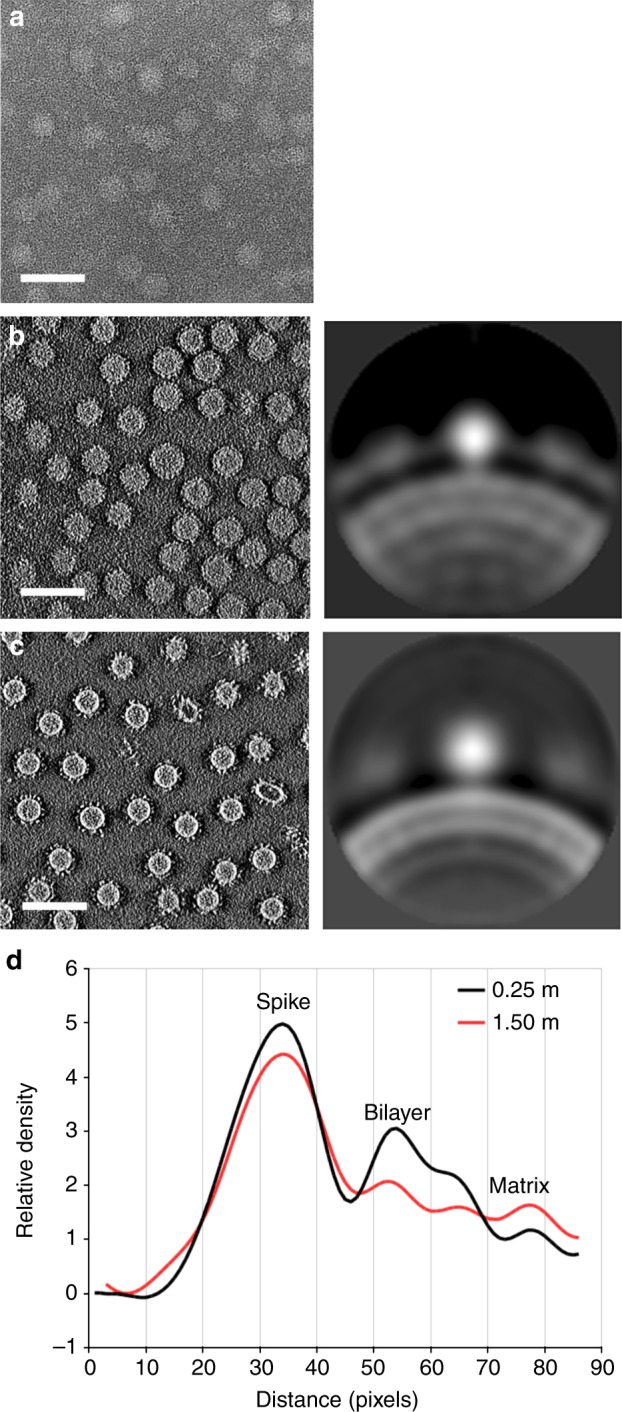
Effect of NaCl concentration and the Volta-phase plate on the tomographic data. **a** A slice through a 3D tomogram of HRPV-6 in 1.5 M NaCl buffer collected without Volta-phase plate. **b** A slice through a 3D tomogram of HRPV-6 in 1.5 M NaCl buffer collected with Volta-phase plate (left), and the corresponding cross section of cylindrically average spike and density distribution (right). **c** A slice through a 3D tomogram of HRPV-6 in 0.25 M NaCl buffer collected with Volta-phase plate (left), and the corresponding cross section of cylindrically average spike and density distribution (right). **d** Averaged radial density distributions of HRPV-6 in low (0.25 M NaCl) and high salt (1.5 M NaCl) are compared. The radial density distribution for the spike is similar at low- and high-salt concentration. The scale bar corresponds to 100 nm. Source data are provided as a Source Data file

To determine the low-resolution structure and oligomeric state of VP5 on the HRPV-6 surface, we used cryo-electron tomography and sub-tomogram averaging (Fig. 2a, b; Supplementary Figure 3; Supplementary Table 1). This was facilitated by Volta phase plate technology^10^, which provided sufficient contrast in the tomographic reconstructions for identification and alignment of the relatively small VP5 (~57 kDa) spikes (Figs. 2a and 3). Sub-tomogram averaging of 8953 manually picked spikes resolved the VP5 structure in situ at 16-Å resolution, as estimated by Fourier shell correlation (0.143 threshold). Each virion (*N* = 247) had 35 ± 7 VP5 spikes on the surface and each spike is a monomer with the characteristic triangular shape reminiscent of that seen in the high-resolution X-ray structures (Fig. 2c, d). In cryo-electron microscopy, high concentrations of salt surrounding the particles diminish the contrast, hence HRPV-6 virions had been quickly diluted before vitrification. In control experiments, HRPV-6 data collected without reducing the salt concentration showed that the radial profile of the cylindrically averaged spikes has the same height in high and low salt conditions (Fig. 3). Since there is robust agreement between the high-salt X-ray structure and the low-salt tomographic data, transient exposure to low-salt conditions has no major effect on the spike conformation.

We fitted the VP5 X-ray structure into the 16-Å resolution VP5 tomographic density as a rigid body (Fig. 2c). Although the fitting is generally robust (cross-correlation coefficient 0.84), there is additional density in the groove between the N- and C-terminal domains, which slightly protrudes from the EM density (Fig. 2c), suggesting that VP5 attached to the viral membrane may adopt a more compact conformation than in the crystal. The C-terminus is proximal to the viral membrane, consistent with the predicted C-terminal transmembrane region anchoring VP5 to the membrane. Density corresponding to the last 39 residues of HRPV-6 VP5 is not observed: the 23 residues preceding the very last two C-terminal asparagine residues correspond to the TM domain, and the preceding 19 missing residues (glycine rich; Supplementary Figure 1) would act to link the ectodomain to the TM domain. In this model, rigid body fitting placed Val461 close to the membrane but this assertion remains to be validated experimentally. The variable, putative receptor binding N2 subdomain is located at the top of the spike. The accurate localization of the putative fusion peptide was hampered by the relatively low resolution (16 Å) of the tomographic reconstruction. Further studies at higher resolution are required to determine how the putative fusion peptide is shielded from solvent in this pre-fusion conformation on the virion surface.

### VP5-spikes induce fusion with host cell membranes

**Fig. 4:**
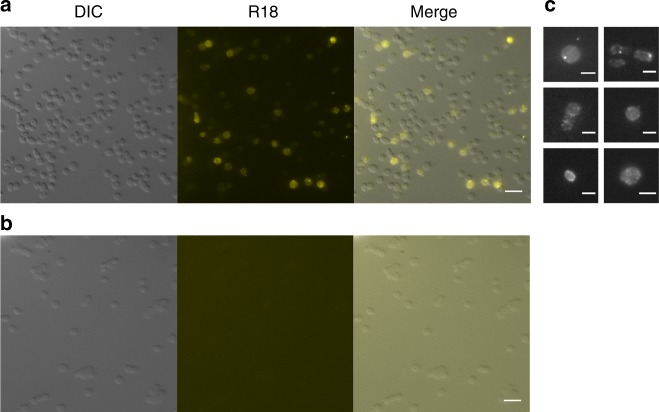
Microscopy of virus-cell fusion assay. Fluorescence microscopy of the *Halorubrum* sp. SS7-4 cells infected with fluorescently labelled HRPV-6 (**a**) or fluorescently labelled spikeless HRPV-6 particles (**b**). **c** A close-up of cells infected with HRPV-6 showing spreading of fluorescence evenly along the cell and viral particles fluorescing brightly. Pictures are representatives from at least three separate experiments all of which contained both treatments. Scale bar 5 μm (**a**, **b**) and 2 μm (**c**). DIC, cells imaged using differential interference contrasting; R18, cells imaged using fluorescence for rhodamine label; merge, an overlay of the two channels used. Source data are provided as a Source Data file

With the aim to test whether pleomorphic archaeal viruses fuse their membrane envelope with the host cytoplasmic membrane of cells we established a lipid mixing assay by using haloarchaeal pleomorphic virus HRPV-6 labelled with a lipophilic dye, octadecyl rhodamine B chloride (R18). Labelled viruses were added to the host cell culture for fluorescence microscopy. Cells displayed areas of diffuse fluorescence, indicating that the dye had spread from the viral membrane into the cell membrane (Fig. 4). In some cells, punctate fluorescent signals were also observed. These signals most likely corresponded to virions that were bound to the cell surface, but had not yet made their way through the S-layer and reached the cell membrane (Fig. 4c). As a control, we used labelled HRPV-6 particles, which were treated with proteinase K to proteolytically cleave and remove the spikes, but not the smaller (14.5 kDa) membrane associated protein termed VP4 in this virus. When these spikeless particles were used in equal numbers (as measured by the total R18 fluorescence signal), no fluorescent signal was observed in the cells (Fig. 4b). This verified that viral surface spikes are required for transfer of fluorescence from virions to the host cell membrane.

**Fig. 5:**
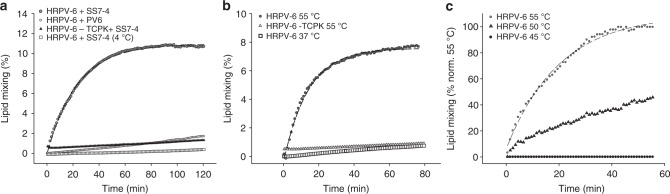
*Halorubrum* virus membrane fusion. **a** HPRV-6 fusion kinetics with cells. Fluorescently labelled HRPV-6 was incubated at 37 °C with *Halorubrum* sp. SS7-4 host cells (grey dots) or the non-host cells *Halorubrum* PV6 (white dots) at 37 °C under continuous stirring and dequenching measured at 585 nm. As additional controls, HPRV-6 was incubated with *Halorubrum* sp. SS7-4 host cells at 4 °C (white boxes) or spikeless HRPV-6 was prepared by proteinase K digestion (TCPK) and incubated with the same host cells at 37 °C (black triangles). **b** HPRV-6 fusion kinetics with liposomes derived from haloarchael SS7-4 cell lipids. Fluorescently labelled HRPV-6 was incubated with liposomes at 37 °C (white boxes) and 55 °C (grey dots). As a control, spikeless HRPV-6 was incubated with haloarchaeal liposomes at 55 °C (white triangles). Results from A and B are representative for *n* = 3 independent experiments. **c** Lipid mixing kinetics of fluorescently labelled HRPV-6 particles after incubation with liposomes at 45 °C (black dots), 50 °C (white triangles) and 55 °C (grey dots) normalized to the maximum extend of fusion at 55 °C. Each segmented line represent the single exponential fit to the kinetic data. Source data are provided as a Source Data file

In vitro time resolved fusion assays based on fluorescent lipid probes have been widely used to characterize fusion kinetics of eukaryotic viruses^11^. To establish such an assay for haloarchaeal viruses, we mixed R18-labelled HRPV-6 particles with cells and monitored R18 de-quenching over time. When we incubated HRPV-6 with its host cells *Halorubrum* sp. SS7-4, lipid mixing over 10% could be observed at 37 °C, revealing a half-time for fusion of 18.1 ± 1.8 min (*n* = 3, Fig. 5a, Supplementary Table 2). To corroborate the specificity of the lipid mixing assay, various control experiments were performed (Fig. 5a). Lipid mixing did not occur when HRPV-6 was incubated with the host cells at 4 °C, confirming the arrestment of membrane fusion. We further found that lipid mixing did not occur with proteinase K digested spikeless HRPV-6 particles, hence indicating that fusion was induced by the viral VP5 spike protein (Fig. 5a). Finally, we found that lipid mixing also did not occur when HRPV-6 was incubated at 37 °C with *Halorubrum* sp. strain PV6, the host of HRPV-1, suggesting the requirement of specific host factors to induce membrane fusion at 37 °C.

After having established the specificity of the kinetic lipid-mixing assay between HRPV-6 and host cells, we proceeded to develop a cell-free fusion assay. To this end, we prepared liposomes from the *Halorubrum* sp. SS7-4 host cells and monitored HRPV-6-liposome fusion at 37 °C. As expected from the inherent removal of host proteins during lipid extraction, no lipid mixing could be observed under this condition (Fig. 5b). Lipid mixing could be readily detected when we increased the temperature to 55 °C, reported to decrease HRPV-1 infectivity by partial denaturation^4^, with a half time for fusion of 12.1 ± 4 min (*n* = 3, Fig. 5b, Supplementary Table 2). This was not the case when spikeless HRPV-6 particles were incubated at 55 °C with the liposomes, confirming that VP5 protein was required for the merger of membranes. To further characterize the temperature of heat-induced fusion, we examined fusion temperatures between 37 °C and 55 °C. Lipid mixing was not detected in the temperature range from 37°C to 45 °C while at 50°C some membrane fusion occurred, albeit at a much lower level than at 55 °C (Fig. 5c). These data together confirm that HRPV-6 infects cells by virus-cell membrane fusion induced by its VP5 spike protein. Although host cell infection and fusion seems to depend on stringent host cell factors in vivo, VP5 protein fusion activation can be triggered through heating to 55 °C.

**Fig. 6:**
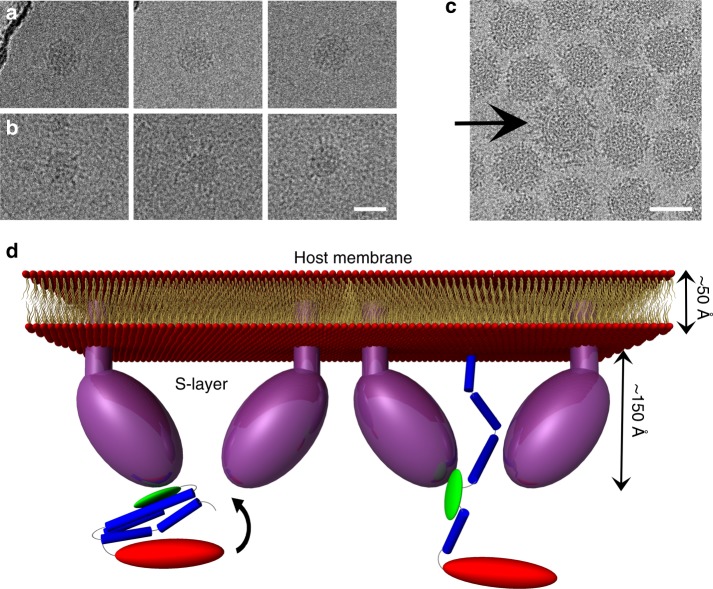
HRPV VP5 extended conformation and proposed model. **a** HRPV-6 virions incubated at room temperature do not show extended spikes. **b** HRPV-6 virions incubated at 55 °C, showing VP5 in more extended conformation. **c** A cryo-EM image showing a particle with extended spikes (low salt). Scale bars correspond to 50 nm. **d** Proposed model for the rearrangement of HRPV spike from the pre-fusion to the membrane insertion step conformation. The N- and C-terminal domains of VP5 are coloured in blue and red, respectively, whereas the putative host recognition (N2) domain is coloured in green. VP5 would bind to the host via its N2 domain and opens up an elongated structure of N1

In order to test whether the heat treatment could trigger a conformational change of the spikes occurring during fusion, we analysed HRPV-6 particles heated at 55˚C for 30 min by cryo-EM. As shown in Fig. 6a, b, we can perceive extended spikes on heat-treated HRPV-6 particles whilst such structures are not observed on particles incubated at room temperature. Because these particles were imaged at high salt, the contrast remains low. Still, a conformational change was observed with spikes of one virion in the sample imaged at low salt conditions (Fig. 6c). The reason for this very rare event of spontaneous triggering is not known. Thus, future studies are required to confirm and further characterise such a conformational change.

## Discussion

A wealth of information describing the mechanisms and the protein structures involved in eukaryotic membrane fusion exists for both cellular processes and infection by enveloped viruses^1^. In contrast, prokaryotic membrane fusion has been studied only in a few cases. One of these is the enveloped bacteriophage ϕ6 which fuses its membrane with the outer membrane (OM) of the Gram-negative host bacterium *Pseudomonas syringae*^2^. The viral protein involved in this fusion process is embedded in the membrane and as such represents an atypical fusion protein with as yet uncharacterized atomic structure. Another class of prokaryotic viral membrane fusion proteins are spanins which are periplasmic proteins and have been suggested to fuse the Gram-negative host cytoplasmic membrane (CM) with the OM consequently facilitating the lysis and exit of the virus progeny from the infected cell^12^. While atomic structures of spanins are lacking, functional analysis of phage lambda spanin suggests similarities to type I fusion proteins^13^.

Our structural analysis of the pleolipovirus spike protein revealed a V-shaped fold dividing the molecule into two elongated domains roughly equal in size. The structure is highly conserved between the two VP5 proteins from two different viruses and released from the virions using two different dissociation methods. HRPV-2 and HRPV-6 VP5 proteins share an average 67% amino acid sequence identity with the N2 subdomain being the least conserved between the two spike proteins. HRPV-2 and -6 infect different host strains suggesting that domain N2 may play a role in host recognition. Furthermore, fitting of the X-ray structure into the HRPV-6 VP5 tomographic density positions N2 subdomain exposed to the surface of the monomer. Thus, we hypothesize that the N2 subdomain is responsible for the host receptor recognition. That question will be the subject of future studies.

Using fluorescence microscopy we were able to show that the lipophilic fluorescent label incorporated in the viral membrane was spreading to the host cell membrane only during infection with the wild-type particles and not with spikeless particles containing the intact smaller structural protein VP4. In concordance with this result, we demonstrated that lipid mixing occurs during the infection of cells by HRPV-6 and that membrane fusion can be induced with host-lipid derived liposomes when HRPV-6 is incubated with liposomes at 55 °C. Such a heat-induced activation based on partial protein denaturation has previously been reported only for class I fusion proteins^14,15^.

In most known haloarchaeal species the cell wall consists of the cell membrane covered by proteinaceous paracrystalline S-layer^16^, a rigid layer which, however, has to be able to accommodate the dynamics of transfer of molecules to and from the cell for cellular responses and maintenance. The pleolipovirus in vivo fusion mechanism may require an active interplay with the host S-layer which most probably harbours the receptor for this archaeal virus. The identity of the host receptor and the trigger for fusion at physiological temperature remain an active area of investigation.

On the basis of the present results, we propose a model for the infection mechanism of haloarchaeal pleomorphic viruses (Fig. 6d). Upon interaction of VP5 with the receptor on the host cell surface, possibly via the N2 subdomain, the α-helical bundle of N1 subdomain would open and extend ~150 Å due to interactions with components of the glycoprotein S-layer. This might further lead to opening of the surface layer allowing projection of the putative fusion peptide towards the host cell membrane (Fig. 6d), reminiscent of the large-scale conformational changes observed in class I viral fusion proteins. The extended conformation would allow VP5 to reach across the ~120 Å thick S-layer^16^. This hypothesis is supported by the evidence of HRPV-6 particles heated at 55˚C where the spikes seem to have switched in a concerted fashion to an extended conformation (Fig. 6a, b). The estimated maximum length of the filament-like spikes (190 Å) also matches our predictions of the maximum length of the extended conformation of the spike (200 Å; Fig. 6d). This preliminary observation of extended conformation of the spikes is intriguing and will be addressed in the future by tomography and sub-tomogram averaging.

Although many aspects of pleolipovirus fusion remain uncharacterized, our results unequivocally highlight the role of VP5 mediating fusion to enter its host cell. Only particles with intact VP5 spikes could fuse their membranes with the host cell and liposome, whereas VP5-free particles could not. VP5 is thus a bona fide fusion protein. The monomeric fold of VP5 on the viral particle surface is unlike all other known viral fusion proteins which fall into one of three classes, I–III^8^. Other likely exceptions to this classification are the still poorly understood and unclassified fusion proteins of hepadnaviruses, poxviruses, pesti- and hepaciviruses^17–20^. We therefore propose that VP5 and related pleolipovirus spike proteins are a different type of fusion proteins, and may be the representatives of a previously unreported class of viral fusion proteins. The unique features of these proteins may reflect their similarity to a very ancient fusion mechanism maintained in Archaea, the evolutionary pressure imposed by the very high salt environment, and the presence of the thick proteinaceous S-layer on the host surface. Further studies on membrane fusion proteins of viruses infecting prokaryotes and eukaryotes may illuminate the evolutionary connections between the fusion proteins of prokaryotic and eukaryotic viruses.

## Methods

### Haloarchaeal host strains and viruses and growth conditions

Haloarchaeal pleomorphic viruses HRPV-2 and HRPV-6 as well as their host strains *Halorubrum* sp. SS5-4 and SS7-4 were initially isolated from samples collected in a solar saltern samples in Samuth Sakhon, Thailand^21,22^ and the non-pigmented derivative of SS7-4 (see below), respectively, were used in this study. *Halorubrum* sp. PV6^5^ was initially isolated from samples collected in a solar saltern in Trapani, Sicily, and used as a non-host strain. Haloarchaeal strains were grown aerobically in Modified Growth Media^23^ (MGM) at 37 °C. MGM broth, plates and soft-agar contained 23, 20 and 18% (wt v^−1^) artificial salt water (SW, HaloHandbook [http://www.haloarchaea.com/resources/halohandbook/Halohandbook_2008_v7.pdf]) respectively. Viruses were propagated on lawns of host cells grown in MGM soft agar. HRPV-2 and HRPV-6 storage solutions were prepared using the top agar of semi-confluent plates and incubating it together with MGM (2 ml for each plate used) at 37 °C with shaking for 2 h. The debris was removed by centrifugation (Sorvall F12, 11,000 × *g* for 40 min at 4 °C) and the cleared lysate was stored at 4 °C. 12% SW-HEPES was diluted from the 30% SW-HEPES stock solution in which Tris-HCl buffer (pH 7.2) was replaced by HEPES buffer of the same concentration and pH.

### Production of non-pigmented mutant strain

Ethyl methane sulphonate (EMS) was used to mutate cells of *Halorubrum* sp. SS7-4 and screen for non-pigmented cells conducted as described by Mevarech and Werczberger^24^. Briefly, cells were grown to exponential growth phase and collected by centrifugation (Sorvall SA600, 9700 × *g* at ambient temperature) followed by resuspension in an equal volume of buffer containing 50 mM Tris-HCl pH 7.2 and 3 M NaCl. EMS (Sigma M0880) was added to the final concentration of 100 µg ml^−1^. Cells were incubated with EMS at 37 °C with aeration for 2 h after which cells were washed twice with the buffer. Finally cells were resuspended in equal volume of MGM broth and grown until they reached the density of ~1 × 10^9^ cfu ml^−1^. Cell suspension was diluted and plated on MGM media plates and grown at 37 °C until colonies appeared.

### Purification of viruses and spike proteins

HRPV-6 and HRPV-2 virions were purified from the storage solutions first by concentrating them with 10% polyethylene glycol 6000 (PEG6000) at 4 °C for one hour with gentle stirring. Precipitated viral particles were collected by centrifugation (Sorvall F12, 11,000 × *g* at 4 °C) and viral pellets were resuspended in 18% SW. Aggregates were removed by centrifugation (Sorvall F14, 11,500 × *g* at 4 °C). To obtain 1× purified virus, concentrated viral solution was subjected to rate-zonal centrifugation (104,000 × *g* at 15 °C for 2 h for HRPV-6 and for 3 h 30 min for HRPV-2, Sorvall AH629) in linear 5 to 20% (wt vol^−1^) sucrose gradient. The 1 × purified virus was further purified with a CsCl gradient (*ρ*=1.3 g ml^−1^) by equilibrium centrifugation (79,000 × *g* for 20 h at 15 °C, Sorvall AH629) to obtain  2 × -purified material that was concentrated by centrifugation (114,000 × *g* for 3 h at 15 °C, Sorvall T647.5). For crystallization of the spike proteins, two methods were used to dissociate and purify the proteins directly from highly purified virions (2×-purified virus). For HRPV-2 VP5 protein, highly purified virions (200 µg ml^−1^) were dissociated with Nonidet P40 at a final concentration of 0.1% (v v^−1^) in HRPV-buffer containing 20 mM Tris-HCl pH 7.5, 1.5 M NaCl, 100 mM MgCl_2_ and 2 mM CaCl_2_. Soluble VP5 proteins were separated from the other dissociation products by rate zonal centrifugation (Sorvall TH641, 210,000 × *g*, 4 h at 15 °C) in linear 5 to 20% sucrose gradient in the HRPV-buffer. Soluble VP5 proteins were collected, concentrated and washed with detergent removal buffer (20 mM Tris-HCl pH 7.5, 0.5 M NaCl) using ultrafiltration (Amicon Ultra Centrifugal Filter Devices, Millipore, 10,000 nominal molecular weight limit). Detergent was removed from the sample using Detergent Removal Spin Columns (Pierce) as instructed by the manufacturer. Purified sample was washed with detergent removal buffer and concentrated by ultrafiltration as described above.

The ectodomain of the HRPV-6 VP5 were released from highly purified virions (2×virus, 200 µg ml^−1^) using proteinase K (Fermentas) digestion at a final concentration of 20 µg ml^−1^ in 12% artificial salt water (SW). After the digestion (3 h, 37 °C) soluble ectodomains of the spike protein were separated from the other dissociation products by rate zonal centrifugation (Sorvall AH629,112,400 × g, 7 h at 15 °C) in a linear 5–20% sucrose gradient in 12% SW. Soluble proteins were collected and concentrated by ultrafiltration (Amicon Ultra Centrifugal Filter Devices, Millipore, 30,000 nominal molecular weight limit) and washed with gel filtration buffer (20 mM Tris-HCl pH 7.2, 1 M NaCl, 40 mM MgCl_2_). The ectodomains of the spike protein was separated from the remaining proteinase K by gel filtration (Superdex 200, HiLoad 16/60, GE Healthcare) and fractions containing the spike protein devoid of proteinase K were pooled, concentrated and washed by ultrafiltration (Amicon Ultra Centrifugal Filter Devices, Millipore, 100,000 nominal molecular weight limit). The buffer of the final protein sample contained 20 mM Tris-HCl pH 7.2, 1.6 M NaCl and 10 mM MgCl_2_.

### Labelling of HRPV-6

Highly purified (2×-purified) HRPV-6 virions produced in the non-pigmented SS7-4 host were labelled using R18 (O-246, Molecular Probes). Approximately 1.7 × 10^13^ plaque forming units per millilitre (PFU ml^−1^) were mixed well with 45 µg ml^−1^ (62 µM) of the lipophilic dye and divided in two aliquots of equal volume. After one hour-incubation at 37 °C in dark, proteinase K (Fermentas) was added to one aliquot at final concentration of 200 µg ml^−1^ and both aliquots were incubated further for another 1 h at 37 °C. Virions were purified by rate zonal centrifugation in a linear 5–20% sucrose gradient (Sorvall TH641, 154,000 × *g* for 3 h 30 min at 15 °C). Light scattering zones containing the virions were collected, concentrated and washed by ultrafiltration (Amicon Ultra Centrifugal Filter Devices, Millipore, 100,000 nominal molecular weight limit). The infectivity of the labelled particles was determined by titration and the incorporation of fluorescent label by measuring the released fluorescence in counts per second (cps) at Ex 560 nm and Em 590 nm in the presence of 0.17% Triton X-100 (v v^−1^) in 12% SW-HEPES buffer using FluoroMax-4 spectrofluorometer (HORIBA Jobin Yvon Inc). Specific infectivity was determined as plaque forming units per counts of fluorescence in one second (PFU cps^-1^) and it was 72 PFU cps^−1^ for the wild-type HRPV-6 particles and 8.0 × 10^−2^ PFU cps^−1^ for the spikeless particles. Analysis of the viral particles in SDS-PAGE showed that most of the spike protein was removed by the proteinase K digestion (Supplementary Figure 4).

### Adsorption tests

The host culture to be tested was diluted to A_550nm_ of 0.1 and grown to an approximate cell density of 2–3 × 10^8^ colony-forming units per millilitre (CFU ml^−1^, A_550nm_ 0.45–0.5) at 37 °C with shaking (~16 h). Cells were collected by centrifugation (Sorvall SA600, 9700 × *g*, 5 min, RT) and resuspended in either MGM or 12% SW- HEPES. Viruses were added to the cell culture at multiplicity of infection (M.O.I.) of 6 × 10^−7^. Samples of 200 µl were withdrawn at indicated time points, cells were collected by centrifugation (Eppendorf Centrifuge 5415D 9300 × *g*, 5 min, RT) and the supernatant and the cell pellet, resuspended in 200 µl of fresh growth medium, were titrated in 100 µl aliquots with host cells as described before^5^.

### Fluorescence microscopy

Non-pigmented haloarchaeal SS7-4 host cells produced in this study (see Production of non-pigmented mutant strain) were grown as described for the adsorption test. To a solution of cells at their logarithmic growth phase (A_550nm_ of ~0.4–0.5, ~1–2 × 10^8^ CFU ml^−1^) labelled viruses were added at an approximate M.O.I. of 10–50 (virions devoid of proteinase K treatment) and approximately the same number of proteinase K-treated viral particles as determined by released fluorescence. Suspensions were incubated at 37 °C for 15 min with shaking after which cells from 1 ml of sample were collected by centrifugation (Eppendorf Centrifuge 5415D, 5900 × *g*, 5 min, RT) and washed once with MGM broth. Cells were incubated for additional 10 min after which the media was changed into 12% SW-HEPES by pelleting and resuspension of the cells twice.

For microscopy, cells in 12% SW-HEPES were fixed with paraformaldehyde (final concentration 5.2% v v^−^^1^) for 20 min and washed with 12% SW-HEPES three times. Aliquots of fixed cell suspension were mounted on 1% agarose in 12% SW-HEPES and visualised using a Leica DM6000B (Wezlar, Germany) microscope with ×63/1.40 HCX PL APO Lbd.bl. oil objective with ×1.25 magnification changer. Final pixel size is 69 nm. Differential interference contrasting (DIC) was used with bright field and Semrock Brightline TRITC-B (ex 543/22, em 593/40) filter to detect the fluorescence of R18. A Hamamatsu Orca Flash 4.0v2 camera was used to capture 16 bit images. 700 ms exposure time was used for fluorescence channel Leica Application Suite X (LAS X) and Fiji ImageJ 1.51n was used for image processing. The fluorescence signal of all obtained images were adjusted to maximal fluorescence of 1200 and within one experiment, images obtained from DIC and fluorescent channels were all aligned manually in the same way.

### Virus-cell and virus-liposome fusion assay

Virus fusion with target membranes was monitored by fluorescence dequenching of R18-labelled virions by standard techniques^25^. To this end, purified (1×-purified) HRPV-6 virions produced in the non-pigmented *Halorubrum* sp. SS7-4 host were labelled with R18 at a self-quenching concentration as described above. R18 labelled HRPV-6 virions were separated from excess probe by using a sephadex G-75 column (GE Healthcare). As a negative control, R18 labelled and purified HRPV-6 particles were treated with proteinase K at 200 μg ml^−1^ for 1 h at 37 °C to eliminate any exposed membrane proteins.

In the case of virus**–**cell fusion, non-pigmented haloarchaeal cells were used at their logarithmic growth phase (OD 0.4–0.5, adsorption at 550 nm). For virus–liposome fusion, total lipids were extracted from non-pigmented haloarchaeal SS7-4 host cells based on one phase alcoholic solvent system based on methanol-chloroform-water (1:2:0.8 v v^‒1^) extraction as described before^26^ and resuspended in HRPV buffer. Fresh liposomes were prepared by the freeze-thaw and extrusion method^27^ using a polycarbonate filter with a pore size of 0.2 mm (Avanti Polar Lipids).

To monitor fusion, R18 labelled HRPV-6 virions (~10^8^ PFU) were mixed with either non-pigmented haloarchaeal cells at a MOI of 10**–**20 or with liposomes derived from SS7-4 host cells (5 mg ml^‒1^) in a fluorimeter cuvette under continuous stirring at the indicated temperatures. Fluorescence dequenching was recorded continuously every 60 s at 585 nm at an excitation wavelength of 565 nm using a fluorescence spectrophotometer (Varian Eclypse, Agilent Technologies) with 5 and 10-nm slit width for excitation and emission, respectably. The base value at time 0 was defined as 0% lipid mixing and the maximal extent of R18 dilution was determined by the addition of Triton X-100 (final concentration 0.1%) after the lipid mixing of each condition had concluded. After data collection, the lipid mixing kinetic was fitted to a single exponential fit using Eq. (1).

1 $${\mathrm{Lipid}}\,{\mathrm{mixing}}\,\left( {t} \right){\mathrm{ = Lipid}}\,{\mathrm{mixing}}_{{\mathrm{max}}} \times {\mathrm{(1 - exp( - \tau }} \times {t}{\mathrm{))}}$$

where *τ* is the time constant and Lipid mixing _max_ corresponds to the maximum lipid mixing value at infinitive time.

### LC-MS/MS

Peptides were quenched with 10% trifluoroacetic acid (TFA) and purified with C18 microspin columns (Harvard Apparatus, USA) eluting the samples to 0.1% TFA in 50% acetonitrile (ACN). The dried peptides were reconstituted in 30 µl 0.1% TFA in 1% ACN (buffer A). Liquid chromatography coupled to tandem mass spectrometry (LC-MS/MS) analysis was carried out on an EASY-nLC 1000 (Thermo Fisher Scientific, Germany) connected to a Velos Pro-Orbitrap Elite/Q Exactive hybrid mass spectrometer (Thermo Fisher Scientific, Germany) with nano electrospray ion source (Thermo Fisher Scientific, Germany). The LC-MS/MS samples were separated using a two-column setup consisting of a 2 cm C18 Pepmap column (#164946 Thermo Fisher Scientific, Germany), followed by 15 cm C18 Pepmap analytical column (#164940 Thermo Fisher Scientific, Germany). The linear separation gradient consisted of 5% buffer B in 5 min, 35% buffer B in 60 min, 80% buffer B in 5 min and 100% buffer B in 10 min at a flow rate of 0.3 µl min^−1^ (buffer A: 0.1% TFA in 1% acetonitrile; buffer B: 0.1% TFA acid in 98% acetonitrile). 4 µl of sample was injected per LC-MS/MS run and analysed. Full MS scan was acquired with a resolution of 60,000 at normal mass range in the orbitrap analyzer the method was set to fragment the 20/10 (QE 10 and OQ top 20) most intense precursor ions with CID (Elite OE/HCD with QE) (energy 35). Data were acquired using LTQ Tune software.

Acquired MS2 scans were searched against home-made protein database using the Sequest search algorithms in Thermo Proteome Discoverer. Allowed mass error for the precursor ions was 15 ppm. And for the fragment in 0.8 Da/or 0.05 Da(Q Exactive). A static residue modification parameter was set for carbamidomethyl +57,021 Da (C) of cysteine residue. Methionine oxidation was set as dynamic modification +15,995 Da (M). The analysis was carried out at the Protein Chemistry Core Laboratory, Institute of Biotechnology, Helsinki Institute of Life Science HiLIFE, University of Helsinki.

### Crystallization and data collection

Both HRPV-2 VP5 and HRPV-6 VP5 proteins were crystallized in 96-well plates (Greiner Bio-One Ltd, Stonehouse, England) using the sitting-drop vapour-diffusion method at 21 °C with a Cartesian Technologies MIC4000 robot^28,29^ (Digilab). HRPV-2 VP5 crystals grew in a solution containing 12% (w v^−1^) PEG 4000, 0.1 M Na-HEPES pH 7.5 and 0.1 M NaCl from the MemSys crystallization screen (Molecular Dimensions). Crystals were first stabilised in the same reservoir solution but containing 30% (w v^−1^) PEG 4K and then dipped into oil (Perfluoropolyether PFO-X175/08, Hampton Research) before flash-cooling in liquid nitrogen. A dataset (space group *P*2_1_2_1_2_1_) was collected on beamline I24 at Diamond Light Source (DLS, Didcot, England) to 2.5 Å resolution (unit cell *a* = 48.1, *b* = 93.2, *c* = 121.8 Å, *α* = *β* *=* γ = 90°). Initial crystals of HRPV-6 VP5 grew in 1.6 M Ammonium Sulphate, 0.1 M Citrate pH 5.0 from the Ammonium Sulphate Grid Screen (Hampton Research) and were further optimized by adjusting the pH between 5.4 and 5.8 and the precipitant between 1.6 and 1.8 M. Due to the high NaCl concentration in the protein buffer, NaCl was added to all the reservoir solutions to a final concentration of 1.6 M. For phasing purposes, HRPV-6 VP5 crystals were briefly immersed in the reservoir solution containing 0.2 M NaBr and 30% (w v^−1^) ethylene glycol prior to flash-cooling in liquid nitrogen. In order to optimize the anomalous signal from the bromine, a dataset was collected close to the bromine edge at 0.9150 Å on the I02 beamline at DLS employing the inverse-beam method. Crystals diffracted to 2.7 Å resolution and belonged to space group *P*6_5_22 with unit cell parameters *a* = *b* = 114.3, *c* = 445.2 Å, *α* = *β* = 90°, *γ* = 120°. HRPV-2 and HRPV-6 VP5 datasets were indexed, integrated, and scaled with XIA2^30–32^.

### Structure determination and refinement

Structure determination of HRPV-6 VP5 via the single-wavelength anomalous dispersion (SAD) method used the anomalous scattering from the bromine atoms. HKL2MAP^33,34^ determined the position of 20 bromine atoms, which were then used in PHENIX.AUTOSOL^35^ for phasing. The experimental electron density map was clearly interpretable and the structure was initially partially built with PHENIX.AUTOBUILD^35^ and then completed by manual building in COOT^36^. Two molecules were present in the asymmetric unit, although no density could be seen for half of one of them. HRPV-2 VP5 (62% sequence identity to HRPV-6 VP5) was solved by molecular replacement using PHASER^37^ using HRPV-6 VP5 as a search model. Both structures were refined with AUTOBUSTER^38^ to *R*_work_ and *R*_free_ of 21.8/23.3% for HRPV-6 VP5, and 22.4/24.1% for HRPV-2 VP5. Final structures were validated with MOLPROBITY^39^ with more than 97% of residues in the Ramachandran favoured regions and no outliers. Data collection and refinement details are presented in Table 1, and a stereo view of the final electron density maps are shown in Supplementary Figure 5.

### Protein in silico analyses

The hydrophobic regions of HRPV-2 and HRPV-6 VP5 proteins were predicted using TMpred server^9^, Phobius^40^ and MPEx^41^.

### Sample preparation for electron microscopy

VP5 spikes on HRPV-6 are very sensitive to ionic strength and only stable in 1.6 M NaCl or above. At lower concentrations of NaCl the spikes fall apart. As a result a careful optimisation of NaCl concentration and incubation time was performed. For cryo-EM, samples were prepared by diluting the purified virus (10×) in the buffer containing 250 mM NaCl immediately prior vitrification. Aliquots of 4 μl were added onto a glow-discharged holey carbon copper grid (C-flat, CF-2/1-2C; Protochips). Grids were blotted for 3 s, in 90% relative humidity, and vitrified in liquid ethane with a plunger device (Vitrobot; FEI). In order to keep the spikes intact and in native form, grids were plunged within 30 s of dilution to low salt buffer. To assess the salt concentration effect on the virions and spike, HRPV-6 in 1.5 M NaCl buffer were plunge-frozen on the grids as control.

To study heat-treated particles of HRPV-6, 100 µl of highly purified particles (~1 × 10^12^ pfu ml^−1^) were split into two 50 µl aliquots. One aliquot was incubated at 55 °C and one at room temperature for 30 min in HRPV-buffer. Aliquots of 3 µl were added onto glow-discharged copper quantifoil grids (R1.2/1.3), blotted for 4.5 s and vitrified in liquid ethane with a plunger device (Vitrification robot; Leica).

### Cryo-electron microscopy data collection

Cryo-EM data were collected at the national Electron Bio-Imaging Center (*e*BIC), at the Diamond Light Source on a FEI Titan Krios transmission electron microscope operating at 300 kV. The Krios was equipped with an energy filter (GIF Quantum, Gatan) operating in zero-loss mode with a 20 eV slit and a Volta phase plate (VPP; Thermo Fisher). Single axis tilt series (from ‒45° to +45° with angular increments of 3°) were collected using EPU software (FEI) on a direct electron camera (K2 Summit, Gatan). At each tilt, images were recorded as a movie consisting of eight frames with a total exposure of 1.6 s per tilt and at a calibrated magnification of ×22,222 in a single electron counting mode, corresponding to a pixel size of 2.25 Å. The datasets were collected in focus and the standard autofocusing routine implemented in EPU was used at every tilt angle. Cumulative electron dose was kept constant in all datasets and the irradiated area on the VPP was changed after each tomogram. Images of heat treated particles were collected at the University of Helsinki cryo-EM facility on a Talos Arctica electron microscope operating at 200 kV and using Falcon III detector (Thermo Fisher).

### Image pre-processing and tomogram generation

Drift correction^42^ was used to correct for the electron beam induced motion by averaging eight frames for each tilt. 25 tilt series were aligned by patch tracking in IMOD package^43^. The six best tilt series, which showed consistent contrast, were reconstructed into tomograms with a final pixel size of 2.25 Å. Altogether 247 HRPV-6 virions were picked in Bshow^44^ and extracted from the tomograms using Jsubtomo^45^ for further analysis.

### Sub-tomogram averaging

For initial template generation, extracted virion volumes were low pass filtered to 80 Å using Bsoft^44^. Spikes (8953 in total) were manually picked from the volumes of HRPV-6 using Dynamo^46^. All picked spikes were extracted from the unfiltered tomograms into boxes of 128 × 128 × 128 voxels. Sub-tomogram averaging was carried out in Dynamo^46^, following protocols we have established earlier^45,47^. In the first stage of refinement, both the locations and directions of the picked particles were allowed to change. The angle around the spike long axis (azimuth) was kept fixed. The refinement was carried out while restricting the resolution to 36 Å, during which a large spherical mask (radius 63 pixels) and full cylindrical symmetrization were applied to roughly align the spikes.

For the second stage, a customized post-processing plugin^47^ was designed to carry out gold-standard refinements where only the azimuth angle was refined. The dataset was randomly split into two datasets consisting of 4502 and 4451 particles and each dataset was averaged to produce a template for refinement. Independent refinements were carried out on the two datasets. At the end of each iteration, the Fourier shell correlation (FSC) between the two averages was computed and the resolution estimated using a criterion of 0.143. Two shells in Fourier space were subtracted from the estimated resolution value, and the difference was used as the threshold for a low-pass filter for the next iteration. A spherical mask (radius 32 pixel), encompassing only the spike ectodomain was used, and no symmetry was assumed. After this stage, 1436 particles were removed from the dataset due to overlaps. Averaging the remaining particles produced an ellipsoidal-shaped spike density, which was connected with the membrane by one, possibly two small stalks.

In the third stage, an ellipsoidal mask (semi-principal axes of length 40, 24 and 24 pixels in the *x*, *y* and *z* direction), encompassing only the spike ectodomain was used. In this stage, the azimuth angle was further refined with small angular search steps. After this stage, 629 particles were removed from the dataset due to low cross-correlation. The coordinates of the remaining particles were plotted and 226 particles located obviously outside of the virus were further removed.

In the fourth stage, all six parameters (three location coordinates and three Euler angles) were allowed to change simultaneously. After this stage, 741 particles were removed due to low cross-correlation or overlap, giving finally 3112 and 2945 particles for the two datasets. The final map resolution of 16 Å was estimated by FSC, using 0.143 as threshold. To assess the salt concentration effect on the virions and spike, 1200 spikes from HRPV-6 in 1.5 M NaCl buffer were cylindrically aligned and averaged.

### X-ray structure fitting

X-ray structure of HRPV-6 VP5 was fitted as a rigid body into the segmented EM density of the VP5 spike in UCSF Chimera^48^. Initial manual fitting that placed the C-terminus proximal to the membrane was improved by ‘fit in map’ function (correlation 0.83).

### Reporting summary

Further information on experimental design is available in the Nature Research Reporting Summary linked to this article.

## Supplementary information

Supplementary Information

Reporting Summary

Source Data

## Data Availability

Data supporting the findings of this manuscript are available from the corresponding authors upon reasonable request. A reporting summary for this Article is available as a Supplementary Information file. The source data underlying Figs. 3d, 4, 5, Supplementary Figure 2 and 3b are provided as a Source Data file. Coordinates and structure factors of HRPV-2 VP5 and HRPV-6 VP5 have been deposited in the Protein Data Bank under ID codes 6QGI and 6QGL, respectively. The accession ID for the EM map reported in this paper is EMD-9779 and the PDB-code for fitted HRPV-6 VP5 is 6J7V. The MS/MS data was submitted to PeptideAtlas with identifier PASS01324.
