# Supplementary material for: The structure of a prokaryotic viral envelope protein expands the landscape of membrane fusion proteins
Source: Nat Commun. 2019 Feb 19;10:846. doi: 10.1038/s41467-019-08728-7 (PMC6381117; doi:10.1038/s41467-019-08728-7)
Supplement: Supplementary file 1 — Supplementary Information [file 41467_2019_8728_MOESM1_ESM.pdf]

Supplementary Information

The structure of a prokaryotic viral envelope protein expands the landscape of membrane fusion proteins

El Omari et al.

Supplementary Table 1. Cryo-electron tomography data collection and model fitting statistics.

	HRPV-6 VP5 (EMD-9779) (PDB:6J7V)
**Data collection and processing**	
Magnification	22,222
Voltage (kV)	300
Electron exposure (e–/Å^2)	49.6
Defocus range (μm)	0*
Pixel size (Å)	2.25
Symmetry imposed	C1
Initial sub-tomograms (no.)	8,953
Final sub-tomograms (no.)	6,057
Map resolution (Å)	16
FSC threshold	0.143
**Fitting**	
Initial model used (PDB code)	6QGL
Model resolution (Å)	16
Map sharpening *B* factor (Å^2)	0
Validation	
Cross-correlation	0.84

* Data collected using Volta phase plate at focus

Supplementary Table 2. Kinetic parameters obtained from HRPV-6 fusion with cells or liposomes. Data represents average +/- SD derived from n=3 independent experiments.

	HRPV6-Cell fusion (37°C)	HRPV6-Liposome fusion (55°C)
Lipid mixing $_{max}$(%)	11.7 ± 1.4	7.1 ± 1.7
τ (s^{-1})	2.3 ± 0.2	3. 7 ± 1.0
t1/2 (min^{-1})	18.1 ± 1.8	12.1 ± 4.0

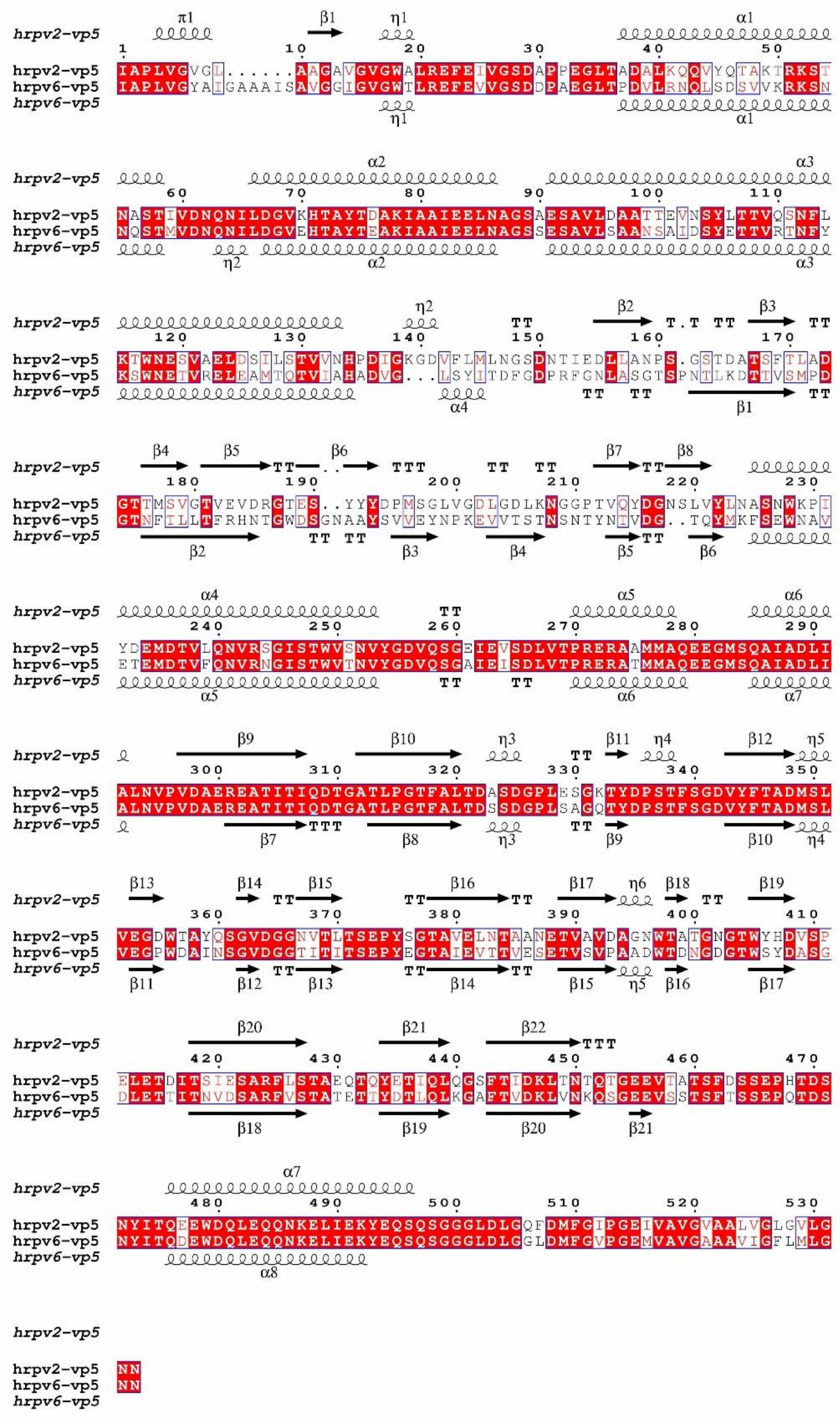

Supplementary Figure 1. | Sequence and structural alignment of HRPV-2 and HRPV-6 VP5. Conserved residues are drawn in red boxes, similar residues in red type. The secondary structure assignment for HRPV-2 VP5 is shown at the top of the sequence and the one from HRPV-6 at the bottom. The alignment was drawn with ENDscript[1].

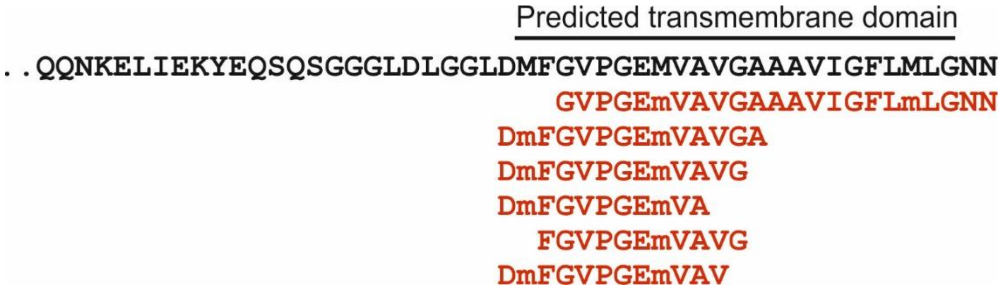

Supplementary Figure 2. | C-terminal peptides of HRPV-6 VP5 determined with LC-MS/MS after proteinase K treatment. The last 50 aa residues of the VP5 C-terminus are shown in black and the peptide sequences obtained using LC-MS/MS (in red) are aligned according to the sequence. The predicted transmembrane domain of the C-terminus was determined using MPex. Source data are provided as a Source Data file.

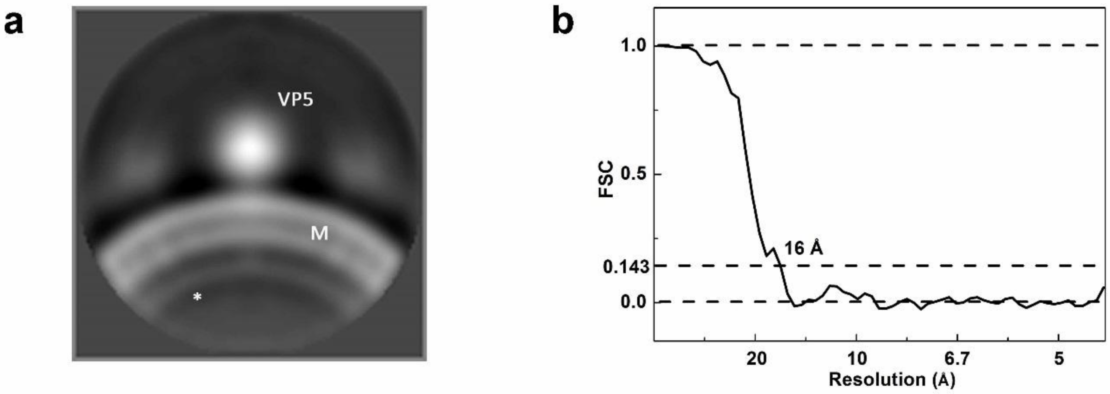

Supplementary Figure 3. | Cryo- EM analysis of HRPV-6 VP5 spike structure. **a** Cross-section of cylindrically averaged spike and density distribution. HPRV-6 VP5 (VP5), the viral membrane (M) and an internal, membrane proximal layer of density (*) are indicated. **b** Fourier shell correlation (FSC) based resolution assessment of the EM map. Source data for FSC is provided as a Source Data file

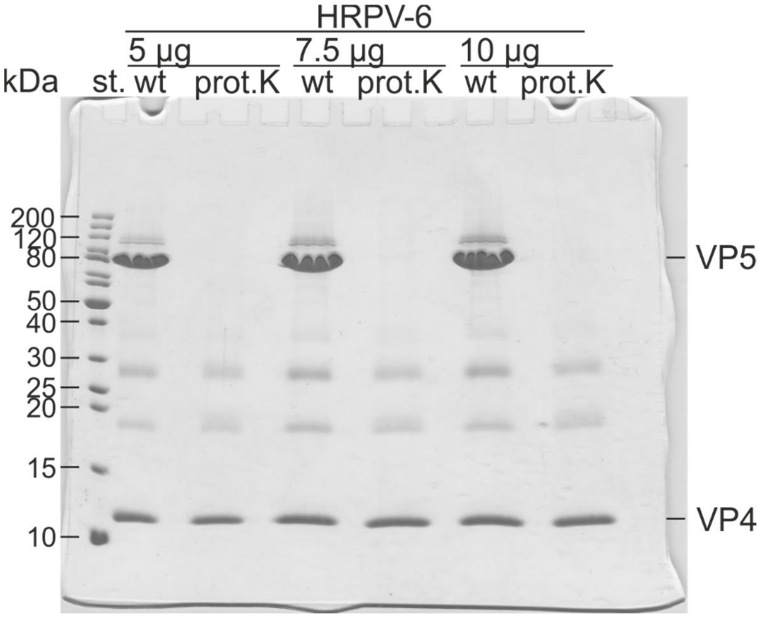

Supplementary Figure 4. | SDS-PAGE analysis of proteinase K-treated HRPV-6 particles. Total amount of viral protein (5, 7.5 and 10 µg) of the undigested HRPV-6 particles was determined by Bradford assay (2). Equal loaded amounts of undigested and proteinase K-digested particles was estimated on the basis of the ssDNA yield obtained when genomic ssDNA was purified from equal volumes of the undigested and digested viral particles in parallel. st., PageRuler Unstained Protein Ladder (Thermo Scientific, #26614); wt, wild type HRPV-6; prot.K, proteinase K-treated particles; VP5, HRPV-6 spike protein VP5; VP4, HRPV-6 membrane associated protein VP4.

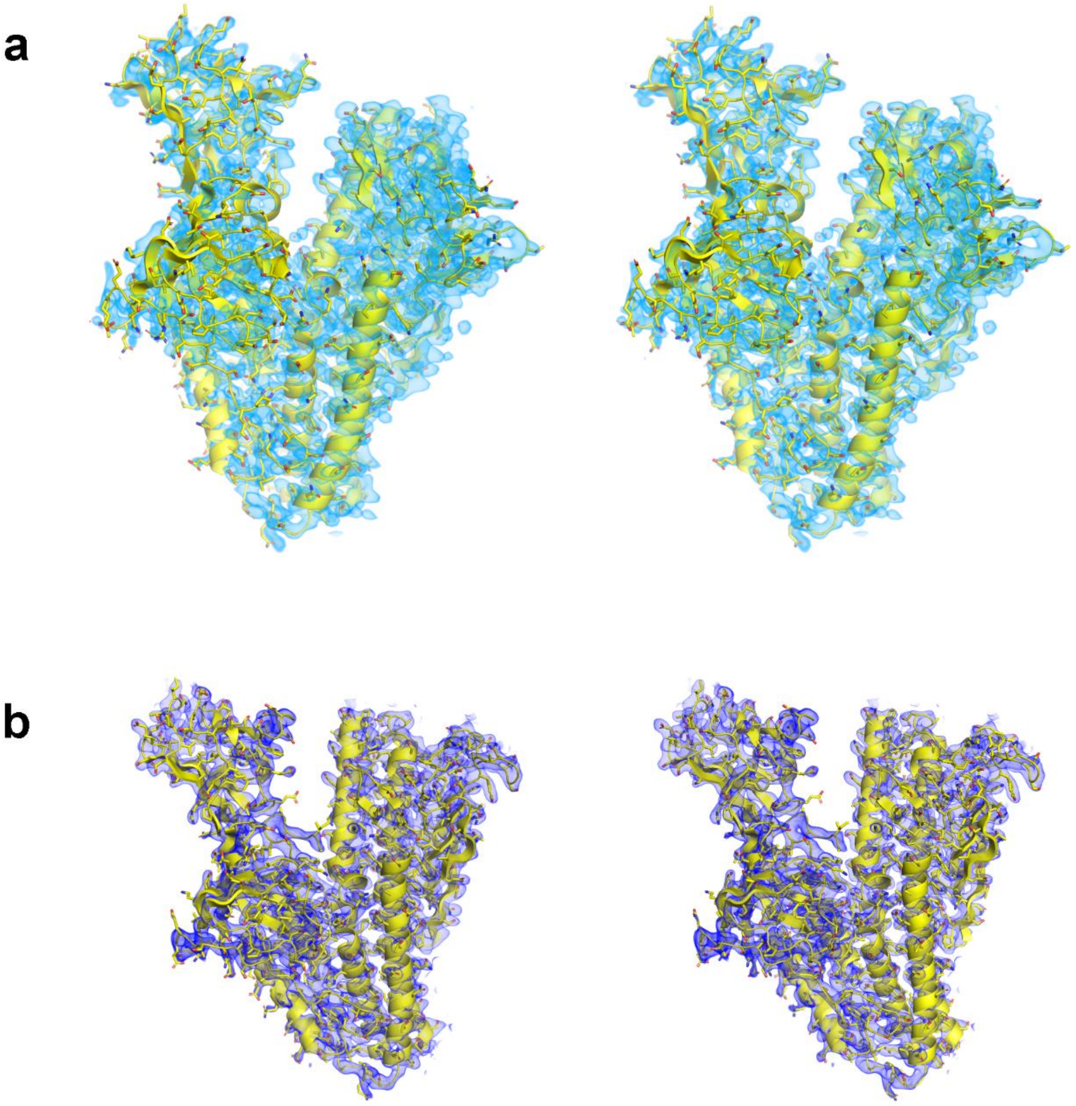

Supplementary Figure 5. | Stereo view of the refined 2Fo-Fc electron density maps of HRPV-6 VP5 (**a**) and HRPV-2 VP5 (**b**) contoured at 1 sigma.

Supplementary References

1. Robert, X. & Gouet, P. Deciphering key features in protein structures with the new ENDscript server. *Nucleic Acids Res.* **42,** W320-324 (2014). doi: 10.1093/nar/gku316.
2. Bradford, M.M. A rapid and sensitive method for the quantitation of microgram quantities of protein utilizing the principle of protein-dye binding. *Anal. Biochem.* **72,** 248-254 (1976).
